# Primary Immunodeficiency May Be Misdiagnosed as Cow's Milk Allergy: Seven Cases Referred to a Tertiary Pediatric Hospital

**DOI:** 10.1155/2013/470286

**Published:** 2013-09-30

**Authors:** Karina Mescouto Melo, Ellen Dantas, Maria Isabel De Moraes-Pinto, Antonio Condino-Neto, Isabela G. S. Gonzalez, Marcia C. Mallozi, Jackeline M. Franco, Beatriz T. Costa-Carvalho

**Affiliations:** ^1^Department of Pediatrics, Federal University of São Paulo (UNIFESP), Rua dos Otonis 725, 04025-002 São Paulo, SP, Brazil; ^2^Department of Immunology, University of São Paulo (USP), Avenida Professor Lineu Prestes, 05508-000 São Paulo, SP, Brazil; ^3^Department of Pediatrics, Federal University of Sergipe, Rua Claudio Batista s/n, 49060-100 Sergipe, SE, Brazil

## Abstract

*Introduction*. The presence of eczema and gastrointestinal manifestations are often observed in cow's milk allergy (CMA) and also in some primary immunodeficiency diseases (PID). *Objective*. To describe 7 patients referred to a tertiary allergy/immunology Center with a proposed diagnosis of CMA, who were ultimately diagnosed with PID. *Methods*. This was a retrospective study based on clinical and laboratory data from medical records. *Results*. Seven patients (6 males) aged between 3 mo and 6 y were referred to our clinic with a proposed diagnosis of CMA. They presented with eczema and/or gastrointestinal symptoms. Five were receiving replacement formula. All patients presented with other clinical features, including severe/recurrent infections unrelated to CMA, and two of them had a positive family history of PID. Laboratory tests showed immune system dysfunctions in all patients. Hyper-IgE and Wiskott-Aldrich syndromes, CD40L deficiency, severe combined immunodeficiency, X-linked agammaglobulinemia, transient hypogammaglobulinemia of infancy, and chronic granulomatous disease were diagnosed in these children. In conclusion, allergic diseases and immunodeficiency are a result of a different spectrum of abnormalities in the immune system and may be misdiagnosed. Educational programs on PID among clinical physicians and pediatricians can reduce the occurrence of this misdiagnosis.

## 1. Introduction

 Primary immunodeficiency diseases (PID) comprise a heterogeneous group of diseases in which there is a defect in the development and/or function of the immune system and to date approximately 150 diseases have been described [1]. Clinical presentation of PID diseases is highly variable, and the hallmark is increased susceptibility to recurrent or severe infections [2, 3]. In addition, some forms of PIDs present with immune dysregulation and lymphoproliferation and others have a more complex phenotype in which infection is only one of the multiple components of the disease phenotype [1, 2].

Cow's milk allergy (CMA) results from an adverse immunological reaction to one or more milk proteins [4]. The clinical signs of CMA are nonspecific and include cutaneous, respiratory, or gastrointestinal manifestations [5]. A detailed history, screening with skin allergy tests, serum specific-IgE (SIgE), and oral food challenge are recommended in guidelines to confirm the diagnosis in IgE-mediated food allergy [5, 6]. A subset of children has non-IgE mediated CMA and present mainly with gastrointestinal (GI) symptoms. However, the symptoms resolve when milk is eliminated from the diet [6]. CMA is one of the most common food allergies in infants. The true prevalence is under discussion; some reports have described a prevalence of 2-3% in children under one year of age and others suggest that it is overestimated [7]. In general CMA is transitory and the majority of children become tolerant by three years of age [7].

Although CMA and PID diseases have different immunological features, similar clinical manifestations can occur in infants, for example, eczema, failure to thrive, and gastrointestinal abnormalities. However, some clues are given by the patients that can differentiate these diseases; particularly in PID, important signs such as recurrent or severe infections are usually observed [2, 8, 9]. The objective of this paper is to present seven children who were initially diagnosed as having CMA but were ultimately diagnosed with PID. The differences between these diseases are also discussed.

## 2. Methods

We selected 7 patients referred to a tertiary allergy/immunology center with a proposed diagnosis of CMA who did not improve after recommended treatment. Data collected from medical records from an allergy and immunology reference center in São Paulo, Brazil.

## 3. Clinical Cases

Seven patients, 6 males, age ranged from 3 mo to 6 y old. The main clinical manifestations and laboratory exams are in Table 1. All patients were full term at birth, only one child was exclusively breastfed for over three months, and family history of atopy was positive in only one patient.

Patient 1 developed an eczematoid rash when he was 3 weeks old, which was related to the introduction of a cow's milk diet. Two months later his eczema had not improved and he presented with a severe pneumonia, requiring hospitalization for 60 days. Over the following years, in addition to persistent eczema, elevated IgE, and eosinophilia, he also had two more severe pneumonias and one lung abscess. When he was three years old, he was submitted to a lobectomy, but the suspicion of PID was raised three years later, after more than 5 years on elimination diet due to chronic eczema.

Patient 2 was a three-month-old boy who was diagnosed with CMA allergy also due to a generalized erythroderma in the neonatal period. The child had two episodes of septicemia during that time. There was a positive family history suggestive of PID, since his oldest brother died at the age of two months due to meningitis and eczema. Laboratory exams with several immunologic abnormalities (Table 1) and absence of thymus shadow in chest X-ray lead to suspicion of severe combined immunodeficiency (SCID), which was confirmed by the presence of very low T and B cell numbers, with normal NK cells in peripheral blood.

Patient 3 was a 1-month-old boy when he started to present with eczema and bloody diarrhea that worsened with infant formula and did not improve with extensive hydrolyzed formula (e-HF). Diagnosis of severe CMA was suspected due to failure of conventional treatment. He was hospitalized twice due to severe infections and petechiae (Table 1). And his laboratory tests showed thrombocytopenia with small platelets, lymphopenia, elevated serum IgE and IgA levels, with normal IgG, and low IgM serum levels that led to a diagnosis of Wiskott - Aldrich syndrome (WAS).

Patient 4 was diagnosed as chronic granulomatous disease (CGD), confirmed by dihydrorhodamine (DHR) test. At two months old she presented with severe diarrhea followed by dehydration and stomatitis. She was admitted to hospital and later transferred to ICU for parenteral nutrition. After 2 months, she was discharged on hydrolyzed formula. She also presented with oral and genital thrush during these first months. Later she developed an anal abscess, eczema, and septicemia and was referred to our clinic when she was 7 months old. Due to the severe infections PID was investigated.

CMA diagnosis was initially presumed in patient 5 when he was three months old due to untreatable diarrhea, gastroesophageal reflux, and failure to thrive. He received replacement formula from that age until 7 months old without improvement. He had a severe pneumonia at 5 months old, and two months later he was hospitalized for 50 days due to septicemia by *Pseudomonas aeruginosa*. Laboratory tests revealed low levels of IgG and IgA, with normal IgM, poor response to vaccine antigens and absence expression of CD40-ligand on activated T lymphocyte leading to the diagnosis of CD40L deficiency or X-linked hyper-IgM (HIM).

Patient 6 is an X-linked agammaglobulinemia (XLA) patient. He was first diagnosed as CMA due to eczema and failure to thrive. As in the other patients his symptoms persisted with replacement formula and after one episode of pneumonia, serum immunoglobulin levels were done, showing a severe reduction in all isotypes. Markedly reduced numbers of B cells confirmed the diagnosis of XLA in this case. Intravenous immunoglobulin was recommended with improvement of his clinical features.

Patient 7 at 1 month old presented with severe bloody diarrhea followed by a septicemia. Serum IgE, IgE to cow's milk, and albumin were normal, but cow's milk was withdrawn from his diet. Protein electrophoresis showed absence of gamma globulin fraction, immunoglobulin levels showed marked reduction of IgG with normal IgM, and he also had normal B cell numbers. To exclude HIM, analysis of CD40L expression was performed and no alteration was found. Intravenous immunoglobulin (IVIG) was recommended with a good outcome after six-month therapy. Parents decided to discontinue IVIG and currently (3 years old) he is still under clinical observation without medication and his immunoglobulin serum levels are normal. Transient hypogammaglobulinemia of infancy was diagnosed and patient's diet is normal.

Following PID diagnosis, IVIG replacement was recommended for all but one patient (P4). Bone marrow transplant was successfully realized in P2 and P4. Unfortunately patient 3 with WAS passed away due to central nervous hemorrhage while awaiting a bone marrow donor transplant.

All seven patients presented recurrent or severe infections but initially the physicians did not consider this a relevant fact. They focused mainly on eczema and gastrointestinal symptoms. Elimination diet was the criterion used to confirm or not CMA diagnosis. Soy formula, amino acid formula, and e-HF were used in five cases. The other two patients were only on elimination diet. None showed improvement with the recommended treatment, so they were referred to our allergy-immunology clinic.

## 4. Discussion

The diagnosis of CMA by pediatricians in these children was based on the clinical picture: eczema, gastrointestinal manifestations, and failure to thrive [4–6]. Except in three children, neither family history nor laboratorial tests were used as parameter to confirm the diagnosis. In these three cases, the complementary test showed eosinophilia and elevated IgE serum to justify the cow's milk free diet. S-IgE to cow's milk was negative in all tested cases, and oral challenge was not performed in any case.

The presence of eczema and gastrointestinal manifestations such as chronic diarrhea and gastroesophageal reflux, associated with failure to thrive, are commonly seen in infants with mixed or non-IgE mediated CMA (Table 2). They include allergic proctocolitis, anaphylaxis, Heiner's syndrome, eosinophilic esophagitis, and food protein-induced enteropathy syndrome. In these cases, patients have normal IgE and the diagnosis is based on resolution of symptoms when the causative food is eliminated from the diet [6]. Except for patient 2, who had a positive family history of PID, the first diagnosis of CMA could be justified according to the initial clinical features. However, two key points were present in our patients to exclude CMA diagnosis during the followup: GI symptoms and eczema did not improve after elimination diet and all seven patients had been hospitalized due to severe infections.

Skin manifestations can affect 40% to 70% of patients with PID younger than seventeen years of age, and in a significant number of them, they are one of the first symptoms [10–13]. they are used as diagnostic criteria for HIES, WAS, immunodysregulation, and polyendocrinophathy enterophathy X-linked syndrome (IPEX). The eczema is usually early, widespread and does not improve with usual medication, as we observed in our patients [14–16]. Gastrointestinal tract involvement can affect more than 50% of patients with PID [17] and it can be secondary to infections, inflammatory or autoimmune diseases, and malignancy [17–19].

Patient 1 was submitted to a long term dietary elimination of cow's milk. This patient fulfilled criteria for hyper-IgE syndrome (HIES) according the NIH proposed score [16]. He had elevated IgE serum, eosinophilia, eczema, recurrent staphylococcal skin abscesses, pulmonary infections, and skeletal abnormalities [16, 20]. Severe atopic dermatitis is the most common disease presenting with eczema and increased serum IgE levels. However, invasive infections are rare in atopic dermatitis and should prompt an evaluation for immunodeficiency [20].

SCID is the most severe form of PID and was diagnosed in P2. His clinical history associated with a careful interpretation of routine exams such as CBC and chest X-ray leads to suspicion of SCID, which is characterized by early onset of infections [21, 22]. Erytroderma with diffuse alopecia is often related to SCID and considered a sign of maternal T lymphocytes engraftment by transfusions of nonirradiated blood [21, 23].

WAS, which is a severe X-linked immunodeficiency, is caused by mutations in *WAS protein (WASP)* gene; the clinical manifestations include eczema, immunodeficiency, thrombocytopenia with small platelets, and an increased risk of developing autoimmunity and malignancies [15]. Eczema affects approximately 80% of all patients; the severity and persistence of it are variable and the cause is still unknown, but allergies could be present in these patients [15, 24]. The patient with WAS (P3) had two previous hospitalizations, one in intensive care therapy (ICT) and after discharge he was advised to continue treatment for severe food allergy. Early diagnosis is crucial to the followup as the life expectancy of patients affected by severe WAS is reduced, unless they have a successful bone marrow transplantation [24].

CGD is a disease characterized by defects in superoxide-generating nicotinamide adenine dinucleotide phosphate (NADPH) oxidase of phagocytes, leading to susceptibility to severe bacterial and fungal infections with exuberant inflammatory responses resulting in granuloma formation in multiple organs [19, 25]. The presence of dermatitis or subcutaneous abscess was the second most frequent clinical finding in an Italian cohort of CGD patients [25]. After CGD diagnosis, P4 was treated with immunosuppressive drugs for inflammatory bowel disease, but she only showed improvement after bone marrow transplantation.

Patient 5 had impaired expression of CD40 ligand on activated CD4+ T cells and also neutropenia, which are common findings in this PID. Gastrointestinal symptoms, pneumonia, and failure to thrive were the initial symptoms, which were all related to CMA although no specific lab exams suggested this diagnosis. Pneumonia and infectious diarrhea are typical clinical pictures of CD40L deficiency, which requires IVIG replacement and appropriate antibiotics [26].

XLA is caused by the mutation of bruton's tyrosine Kinase (*BTK*). Patients present with a marked reduction in all serum immunoglobulin isotypes with significantly decreased or absent B cells in peripheral blood and a lack of specific antibody production, which causes susceptibility to recurrent and severe bacterial infections [1, 27]. Association between XLA and eczema, as observed in patient 6, is not often described but there are reports showing that it can affect some patients and the cause is unknown [28].

Patient 7 with hypogammaglobulinemia was investigated for PID three months after his symptoms started. Suspicion was raised after he suffered from bloody diarrhea followed by septicemia. Except for the low levels of IgG and IgA, the other tests performed to evaluate PID and CMA were normal. Hypogammaglobulinemia secondary to CMA in children was described by Bezrodnik et al., 2007 [29]. In their patients faecal *α*-1-antitrypsin was elevated in 4/5 patients, leading to hypogammaglobulinemia, and in all five cases the patients had high levels of total IgE and S-IgE. Unfortunately in our case, *α*-1 antitrypsin was not performed to evaluate faecal loss of protein, but the patient had normal serum albumin, low serum IgE, and elimination diet alone was not sufficient to improve the clinical features. His immunoglobulin levels increased after his first birthday, suggesting transient hypogammaglobulinemia of infancy.

PID and CMA represent two different spectra of dysfunctions in the human immune system. Both can affect children at an early age; their treatment is totally different and usually involves high emotional and economic issues. A delay in PID diagnosis is usual, and although PIDs are often described as rare disorders, the true incidence is not known and seems to be more common than what was previously thought [30]. In Brazil, the underdiagnosis of PID is a reality [31]; on the other hand, CMA seems to be overdiagnosed, resulting in an important number of children on elimination diets [32]. These unnecessary overprescribed diets are based on a misdiagnosis and may lead to malnutrition and additional psychological stress in patients [4, 32, 33].

In Brazil replacement formula is provided free by the government, we can interpret that this influences the overdiagnosis of CMA in our country [32]. The incorrect treatment of children with PID diseases results in a high rate of sequels and educational programs on PID among health professionals can reduce the delay in PID diagnosis and also reduce the cost of the health system [34].

In conclusion, this paper is a reminder to clinical physicians, allergists, and pediatricians that PID diseases should be suspected when patients present with allergy symptoms associated with severe or recurrent infections. 

## Figures and Tables

**Table 1:** Main clinical manifestations and laboratory tests of children studied.

Patient	P1	P2	P3	P4	P5	P6	P7
Gender	M	M	M	F	M	M	M
Age—onset of symptoms	1 mo	1 mo	1 mo	2 mo	2 mo	3 mo	1 mo
Age—diagnosis of PID	6 y 5 mo	3 mo	6 mo	7 mo	11 mo	1 y 11 mo	4 mo
Clinical features of PID	Eczema Pneumonias Septicemia Lung abscess	Eczema Pneumonias Meningitis Septicemia	Eczema Pneumonias Petechia Meningitis Chronic diarrhea Bloody diarrhea Septicemia	Eczema Severe diarrhea Anal abscess Septicemia	Pneumonias Diarrhea Septicemia Failure to thrive	Eczema Pneumonia Diarrhea Failure to thrive	Neonatal septicemia Bloody diarrhea
Signs of suspicion of CMA	Eczema	Generalized eczema	Vomiting Eczema	Generalized eczema Severe diarrhea	Diarrhea Failure to thrive Gastroesophageal reflux	Eczema Failure to thrive	Bloody diarrhea
Eosinophils/ *μ*L	**747**	**15872**	**742**	170	**880**	159	366
Neutrophils/ *μ*L	1460	4464	6360	5780	704	4526	6100
IgG (g/L)^∗ ^	6.85 (nl)	**0.59** (<p3)	6.26 (nl)	**22.10** (>p90)	**0.19 **(<p3)	**0.78 **(<p3)	**1.46 (<p3)**
IgM (g/L)*	0.73 (nl)	**0.18** (<p3)	0.2 (<p10)	0.86 (nl)	0.47 (nl)	**0.14 **(<p3)	0.51 (nl)
IgA (g/L)*	0.12 (nl)	0.12 (nl)	**3.17 **(>p90)	1.86 (nl)	**0.03 **(<p3)	**0.03** (<p3)	0.06 (<p3)
IgE (IU/mL)	**13000**	**>3000**	**>2000**	<1.5	<2	<1	3.62
S-IgE to cow's milk	Negative	NA	NA	Negative	Negative	Negative	Negative
CD3/*μ*L	2284 (nl)	**597** (<p10)	**827 **(<p10)	4918 (>p90)	3164 (nl)	2050 (>p90)	3382 (nl)
CD4/*μ*L	1244 (nl)	**562** (<p10)	**682** (<p10)	3747 (nl)	2015 (nl)	**808** (<p10)	2541 (nl)
CD8/*μ*L	967 (nl)	**21 **(<p10)	**123 ** (<p10)	1051 (nl)	918 (nl)	1061 (nl)	658 (nl)
CD19/*μ*L	NA	**35 (<2%)**	NA	NA	1994 (nl)	**37 (<2%**)	972 (nl)
Other tests	—	—	—	DHR	CD40L expression	—	—
PID diagnosis	**HIES**	**SCID**	**WAS**	**CGD**	**CD40L deficiency**	**XLA**	**Hypogammaglobulinemia**

*Immunoglobulin levels: values compared to age-matched controls; p10, p90: 10th, 90th percentiles of age-matched reference value; nl: normal value; NA: not available; DHR: dihydrorhodamine test.

**Table 2:** Important clinical findings in CMA versus PID [3, 6].

	CMA	PID
Respiratory tract	Chronic cough* Wheezing* Dyspnea*	Recurrent sinusitis Recurrent/severe pneumonia Otitis Lung abscess Bronchiectasis

Gastrointestinal tract	Frequent regurgitation Abdominal pain Vomiting Diarrhea Constipation Blood in stool Iron deficiency anemia	Chronic diarrhea Giardiasis Hepatic abscess Crohn's disease like Blood in stool Autoimmune enteropathy

Skin	Atopic dermatitis Urticaria	Eczema Furunculosis Abscess Erytrodermia

General	Failure to thrive	Failure to thrive Positive family history of PID

*Unrelated to infection.
